# Predicting Magnetostimulation Thresholds in the Peripheral Nervous System using Realistic Body Models

**DOI:** 10.1038/s41598-017-05493-9

**Published:** 2017-07-13

**Authors:** Mathias Davids, Bastien Guérin, Matthias Malzacher, Lothar R. Schad, Lawrence L. Wald

**Affiliations:** 1 Computer Assisted Clinical Medicine, Medical Faculty Mannheim, Heidelberg University, Heidelberg, Germany; 2 Martinos Center for Biomedical Imaging, Dept. of Radiology, Massachusetts General Hospital, Charlestown, USA; 3 Harvard Medical School, Boston, Massachusetts USA; 4 Harvard-MIT Division of Health Sciences Technology, Cambridge, USA

## Abstract

Rapid switching of applied magnetic fields in the kilohertz frequency range in the human body induces electric fields powerful enough to cause Peripheral Nerve Stimulation (PNS). PNS has become one of the main constraints on the use of high gradient fields for fast imaging with the latest MRI gradient technology. In recent MRI gradients, the applied fields are powerful enough that PNS limits their application in fast imaging sequences like echo-planar imaging. Application of Magnetic Particle Imaging (MPI) to humans is similarly PNS constrained. Despite its role as a major constraint, PNS considerations are only indirectly incorporated in the coil design process, mainly through using the size of the linear region as a proxy for PNS thresholds or by conducting human experiments after constructing coil prototypes. We present for the first time, a framework to simulate PNS thresholds for realistic coil geometries to directly address PNS in the design process. Our PNS model consists of an accurate body model for electromagnetic field simulations, an atlas of peripheral nerves, and a neurodynamic model to predict the nerve responses to imposed electric fields. With this model, we were able to reproduce measured PNS thresholds of two leg/arm solenoid coils with good agreement.

## Introduction

Peripheral Nerve Stimulation (PNS) is a limiting factor in the use of gradient coils in Magnetic Resonance Imaging (MRI), and potentially limits the translation of Magnetic Particle Imaging (MPI) to humans. In MRI, *gradient coils* are used to create a spatially linear magnetic field which encodes the location of the water being imaged^1^. Since the gradients must be switched rapidly (at 100–2000 Hz) to image quickly, the time-varying magnetic field generates electric fields inside the body which can interact with the nerves. MPI uses *drive coils* that produce an oscillating magnetic field (1 kHz to 100 kHz) to modulate the magnetization of magnetic nano-particles^2^. In both imaging applications, the PNS problem, rather than electromechanical engineering of the gradient and drive coils and power supplies, has become the limiting factor to the size and slew rate of the fields that can be used. Thus PNS currently limits imaging capability for at least a subset of imaging sequences in MRI^3, 4^. Most clinical MRI scanners have monitoring software and hardware to ensure that the switching gradients will not cause painful PNS. The thresholds for these monitors are set empirically.

The minimum magnetic field modulation strength dB switched at rise time dt is a commonly used measure of the PNS stimulation thresholds (note that dt determines the pulse duration of the electric field). The ratio of these two quantities, dB/dt, has been routinely used in MR safety considerations due to its simple determination from input waveforms and the role of dB/dt in generating E-fields. Nonetheless, the use of a strict dB/dt limit poses several limitations. In particular, there is wide variation of the dB/dt values for different coil geometries: the Purdue University Study^5^ (based on 84 healthy subjects) found dB/dt values for y and z gradient coils of 15 and 26 T/s, and furthermore observed wide variation of these thresholds with the subject’s position in the coil, waist size, weight, and age. Other studies have reviewed dB/dt at threshold for body MRI gradients noting that the dB/dt at stimulation ranged from 12.4 T/s to 54 T/s^6^. This likely reflects the fact that although dB/dt is the generator of electric fields, the magnitude of the local electric field is shaped by the geometry of the conductive tissues. This is in contrast to the magnetic field, for which the body is largely transparent at these kHz frequencies. Thus, to accurately characterize the capability of a coil to generate PNS, the pattern of the electric field rather than the magnetic field (or its maximum field modulation, dB/dt) must be investigated.

Despite the importance of PNS as the practical limitation to use these imaging modalities in humans, we are not able to fully incorporate PNS thresholds directly into the system design optimization. Instead, they are considered indirectly by controlling the size of the linear region of the gradient coil. It was found that the PNS threshold parameters (i.e., field threshold amplitude and chronaxie of the stimulation threshold curve) vary in an inverse linear manner with the size of the linear region^6^. The development of a robust method for estimating magnetostimulation would potentially allow PNS to be incorporated more directly into the coil design optimization phase. At present, MRI gradient coils are optimized numerically by simulating the current distribution on the coil former so as to achieve the target magnetic field such as a linear gradient field in MRI^7^. Additional penalty terms are included to reduce energy consumption^8^, improve heat dissipation^9^, and simplify the manufacturing process^10^. In this framework, a PNS threshold predictor or “oracle” may be added as an additional penalty term in evaluating a design, potentially uncovering geometries with significantly increased PNS thresholds. In MPI, PNS has presented a barrier from scaling this technology to human-sized scanners. The ability to calculate PNS thresholds might also be useful in evaluating specific situations where workers are exposed to time-varying magnetic and electric fields. Finally, we note that there are applications where coils are designed specifically to achieve stimulation of nerve fibers, for example *transcranial magnetic stimulation* (TMS)^11^ and *nerve conduction studies* (NCS)^12^ to evaluate the viability of motor and sensory nerves. All these applications could benefit from a realistic PNS threshold simulation framework.

The phenomenon of PNS arises from the interaction of the electric fields created by the coil with the nerve fibers in the human body^13, 14^. If an electric potential gradient is applied to a nerve, the nerve membrane will be charged electrically (depolarization or hyperpolarization). In case of a strong depolarization, an unwanted *action potential* (AP) will be initiated, that results in muscle contractions and sensory perceptions. If the applied potential is increased beyond this initial perception threshold, adverse effects can be generated such as pain, stimulation of the central nervous system (possibly triggering seizures), and cardiac nerve stimulation (possibly causing arrhythmia).

The mechanisms behind action potential generation by applied voltage potentials have been well understood for decades^15–20^. For the magnetostimulation problem, the applied magnetic field is typically known. Based on this magnetic field, many approaches have used analytic or semi-analytic methods in order to describe the induced electric field and its effect on the nerve membrane^21–25^, but analytic solutions are restricted to simple geometries (like a cylindrical geometry) and not applicable to complex structures of differing electrical properties such as those found in the body. Zhao *et al*., So *et al*., and Mao *et al*. studied the electric field pattern in a body model with heterogeneous electrical properties and used the electric field strength as a PNS threshold measure^26–28^. This is likely to mis-estimate PNS thresholds since it does not account for the fiber orientation relative to the electric field or the possible lack of nerve fibers in the peak E-field locations. A more accurate approach to assess the electric fields in the body is to use heterogeneous tissue models in conjunction with nerve membrane models to investigate the mechanism of magnetostimulation^29–31^. In these works, the simulations have been performed based on simplified nerve segments which does not allow for PNS threshold prediction unless it is a priori known that the analyzed segment has the lowest threshold in the body. Very recently, functionalized anatomical models have been introduced by Neufeld *et al*.^32, 33^ where the nerve fibers were extended to study segments of the ulnar and sciatic nerves. While providing valuable insights about different neurodynamic model approaches and the effect of tissue heating on the excitability of nerve fibers, the limited nature of the nerve model precluded the calculation of general PNS thresholds. Comparison to experimental data was limited to these few nerve segments. In order to predict the stimulation thresholds that a person would experience from the magnetic and electric fields created by a specific coil winding pattern, a *complete* model of the peripheral nervous system is needed together with the map of the time-varying E-fields created by the coil within the conductive body model.

In this paper, we utilize a realistic depiction of the peripheral nervous system, labeled with the relevant parameters of each segment’s equivalent circuit model. The PNS model is embedded in a realistic body model of the body tissues, each labeled with its electrical properties. This allows for the calculation of the internal time-varying electric fields experienced by the nerves. Knowing the relative geometry of the induced electric field to the nerve and the nerve’s size and membrane parameters, we show that we can accurately estimate the location and threshold for the first action potentials generated by the applied fields. Increasing the strength of the electric field until an action potential is observed provides a measure of the maximum drive current and voltage that can be applied to the coil without causing PNS. Performing this simulation for different coil designs allows for comparison of the design’s PNS thresholds during the design/optimization stage, prior to coil construction.

## Methods

### Workflow

**Figure 1:**
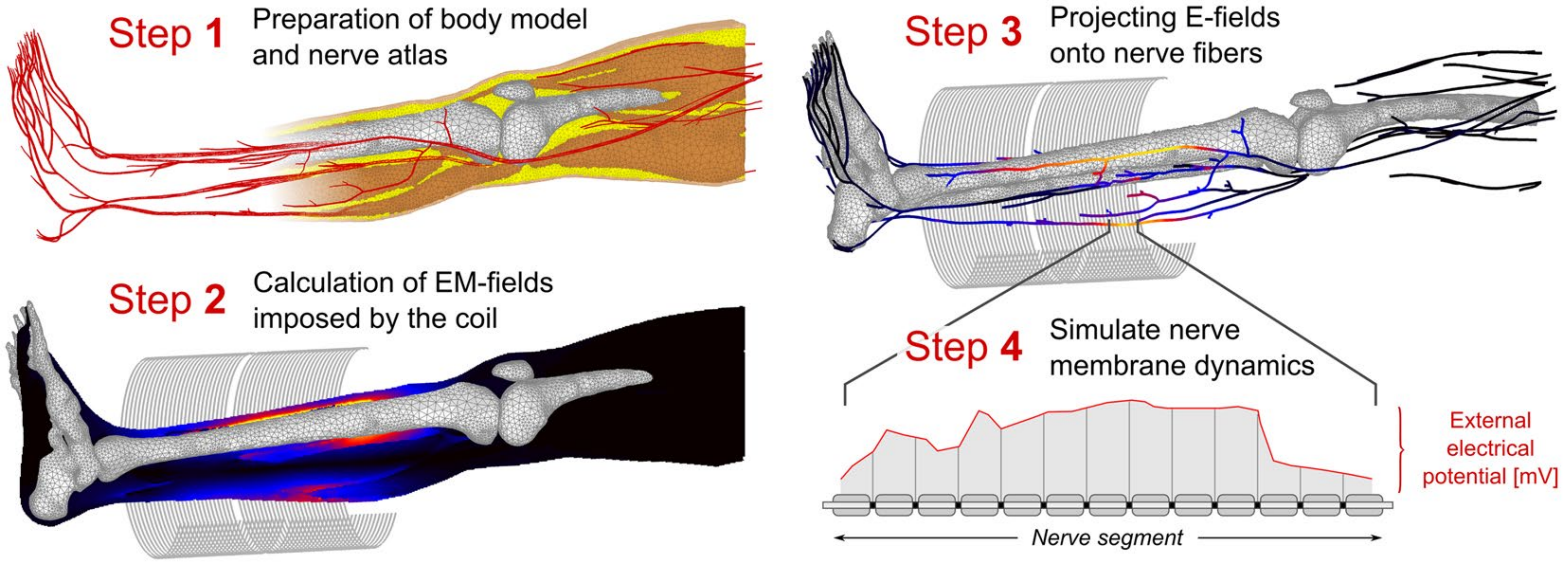
Flowchart of the PNS simulation process. Step 1: Preparation of the body model and nerve atlas (only required once). Step 2: EM fields imposed by the coil are calculated. Step 3: The electric field for a given coil current waveform is interpolated and projected onto the nerve fiber. Step 4: Nerves response is calculated to determine if an AP has been initiated. If no AP is reached, steps 3 and 4 are repeated with a higher amplitude coil current waveform until PNS is observed.

In order to model PNS in the body induced by the switching of electromagnetic fields created by an external coil, we follow the workflow shown in Fig. 1. Our framework has three main components: (A) a surface-based whole body tissue model used for calculation of the internal electromagnetic (EM) fields, (B) a detailed atlas of the nerve fibers in the human body embedded in the same anatomical model, (C) a numerical framework to model the nerve dynamics in presence of external E-fields. In short, we perform the following steps to generate the PNS threshold curves for a given coil geometry:

- We preprocess the surface body model to be compatible with finite element EM field simulations and prepare the nerve atlas to be compatible with the neurodynamic model. This preprocessing step is only required once and can be reused for the analysis of multiple coil geometries.
- We simulate the magnetic and electric fields in the body model generated from a time-varying current applied to the coil using a commercial EM field simulation platform; CST (Darmstadt, Germany).
- The atlas of human peripheral nerve fibers is superimposed on the fields. The simulated electric fields are projected onto the nerves and integrated to obtain the effective electric potential along the fiber. This provides the relevant electric entity that can lead to action potential generation and is used as an input to the *neurodynamic model*.
- The *neurodynamic model* is evaluated providing the response of the nerve fiber to the external electric potentials. The nerve fiber model returns the internal membrane potentials. An action potential (AP) is recognized as a sudden membrane depolarization in response to a small increase in the applied fields.

### Surface Body Model

**Figure 2:**
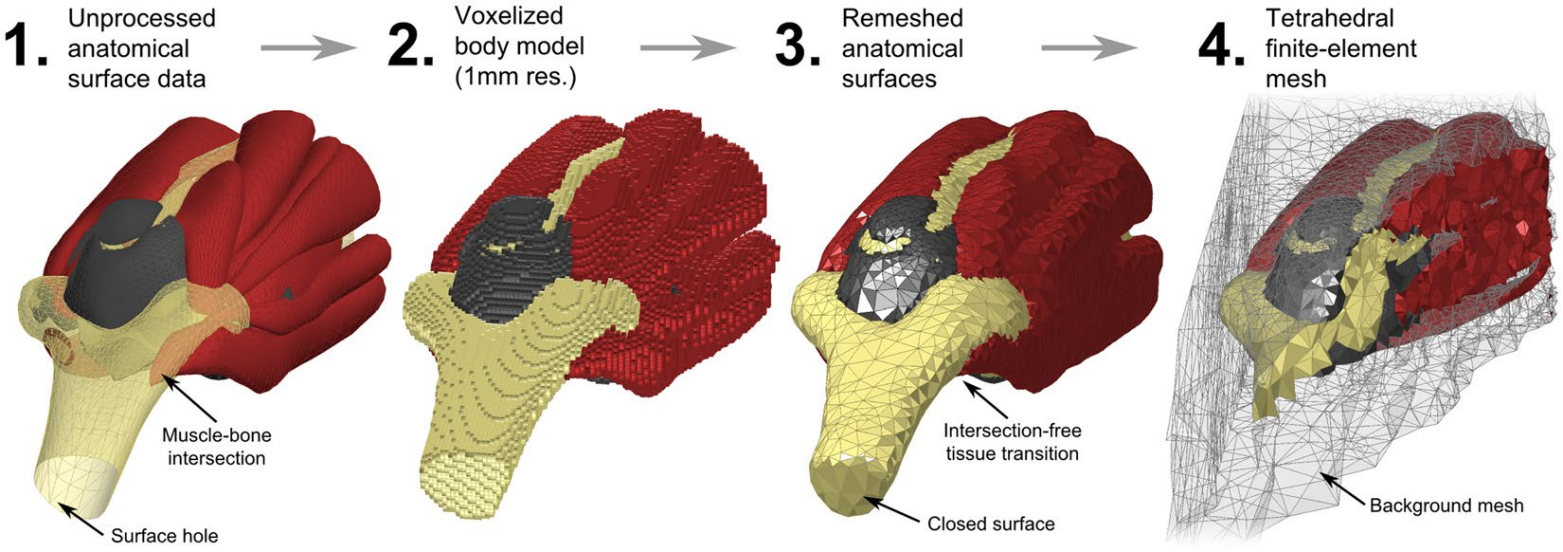
Processing pipeline of an exemplary body model region (elbow region, including muscle, bone, articular capsule): (1) Unprocessed anatomical surface data (provided by Zygote), (2) Discretized (voxel) body model with 1mm voxel size, (3) Watertight intersection-free surfaces (obtained via segmentation of the voxel model), (4) Cross section of an exemplary tetrahedral mesh (computed using CGAL)^35^, similar to the mesh generated by CST Microwave Studio for finite-element EM field simulations.

The realistic body model was based on the anatomical surface data developed by Zygote (American Fork, UT, USA). The Zygote anatomical data includes 12 different tissue classes: skeleton, muscular system, respiratory system, digestive system, nervous system, circulatory system, connective tissue, integumentary system, lymphatic system, urinary system, and endocrine system. Each tissue class is described by a single surface mesh. Since the Zygote model was initially developed for teaching and visualization purposes, further processing was required in order to guarantee that it can be used in conjunction with electromagnetic simulation platforms. Specifically, the input surface mesh to the EM simulation platform (CST AG, Darmstadt Germany) requires an orientable 2-manifold mesh without self-intersections and without intersections of the different tissue classes. To achieve this, we first discretized the surface mesh data to obtain a voxel model at 1 mm isotropic resolution for each tissue class (Fig. 2, Step 1 and 2). The voxels of all tissue classes were combined into a single voxel model. Skin tissue voxels were added by performing a morphological dilation algorithm of the outer body hull, assuming a skin thickness of approx. 3 mm (i.e., 3 voxels). Every interior voxel that was not assigned to any of the tissue classes at this point was defined as fatty tissue (we found that this assumption yields distributions of fatty tissue very similar to well-established body models like the Virtual Population 3.0^34^). This voxelized model guarantees a single label per voxel.

**Figure 3:**
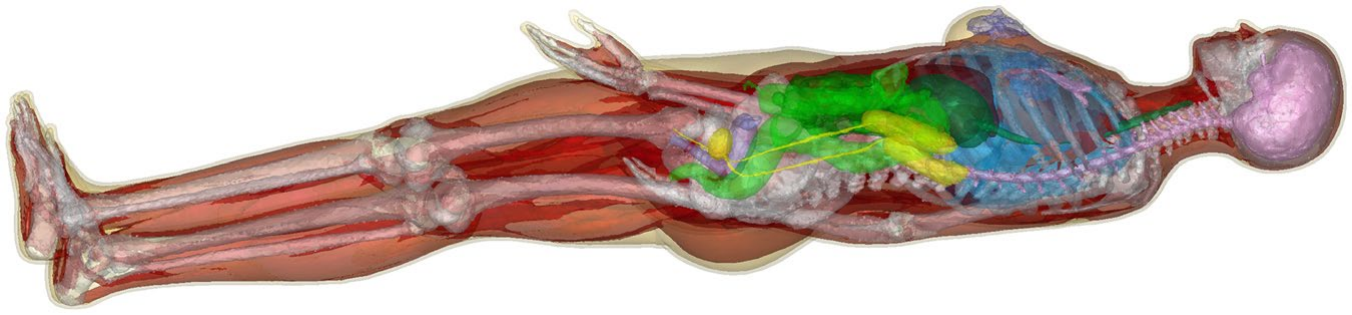
Surface-based whole body model used for the simulation of the EM fields.

We performed a multi-domain surface segmentation of the 1 mm voxel model using the *Computational Geometry Algorithms Library* (CGAL^35^,). The output surface mesh of the segmentation generates a non-intersecting closed surface mesh for each tissue class where all neighboring tissues align perfectly (Fig. 2, Step 3), i.e., neighboring tissues share the exact same faces without creating empty regions or overlaps. However, the resulting surface meshes do not necessarily establish 2-manifold geometries. In the context of surface triangulations, this means that every edge of a mesh is used by exactly 2 faces. Physically, 0-manifold or 1-manifold features of a mesh correspond to physical structures that become infinitely thin or small. Therefore, it is necessary to remove these invalid features from the surface mesh. For that purpose we implemented a method (using Matlab, The MathWorks, Natick, MA, USA) to repair any existing surface mesh error and thus prepare the surface meshes for usage in our EM field simulations. This pre-processing includes elimination of 0-manifold and 1-manifold features, removal of excessively small faces/structures and low-quality faces (i.e., very long and thin faces). In short, this surface mesh repair routine first deletes the faces that correspond to a mesh error. Usually this deletion creates holes in one or more of the tissue surfaces. The routine then identifies possible face configurations to close these holes without causing new surface mesh errors or empty regions. The process is repeated until all surface errors are repaired. An important advantage of this approach over commonly used mesh repair tools is that all tissue surfaces are processed simultaneously in order to ensure that a correction step that is applied to one tissue surface does not introduce intersections or errors in any neighboring tissue surface. Although the creation of the surface body model is computationally intensive (even starting with a commercial meshed model), it only has to be done once per body model. The different tissues were assigned electromagnetic properties (conductivity and permittivity) using the frequency-dependent Gabriel database^36^. Note that the permittivity increases dramatically as frequency becomes very low, and our simulations reflect this. The final surface body model is shown in Fig. 3. It can then be used in an EM field simulation environment (such as CST) in order to generate a tetrahedral finite-element mesh for calculation of the EM fields (an exemplary finite element mesh is shown in Fig. 2, Step 4).

### Nerve Atlas

**Figure 4:**
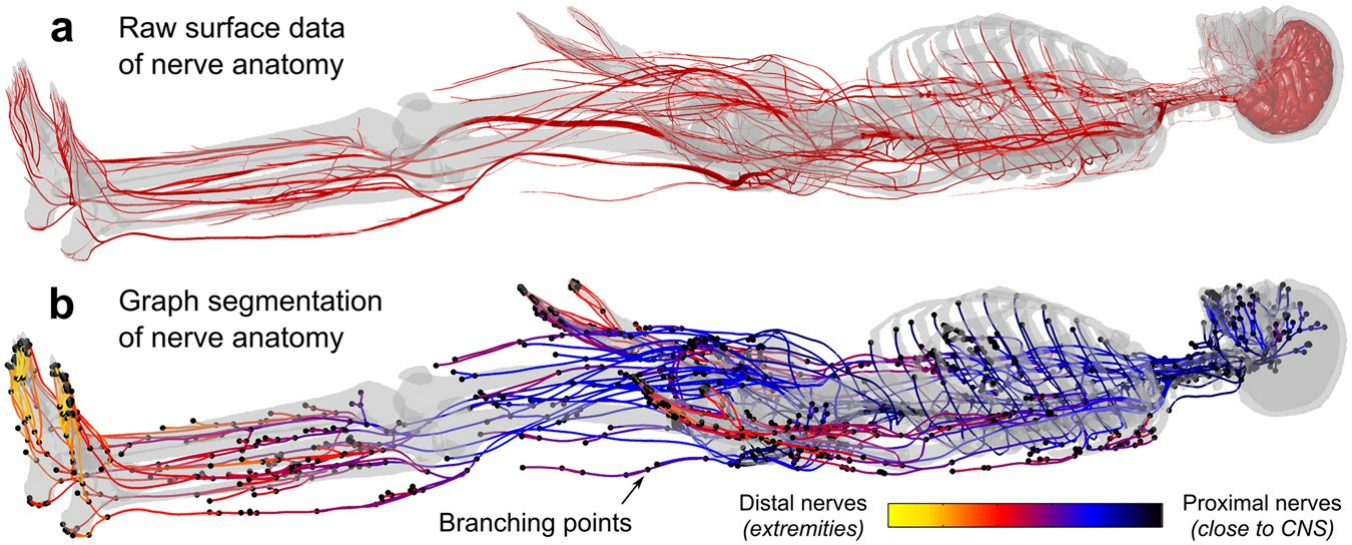
(**a)** Human nervous system model as surface data with skeletal system (transparent gray) for reference. (**b)** Extracted nerve centerlines color coded by distance from the CNS.

The Zygote anatomical model was used, as opposed to more commonly used body models such as the Virtual Family^34^ since the Zygote model includes a detailed atlas of the peripheral nerves. However, like the organ surface mesh data in the model, some processing was necessary to use this nerve atlas information in conjunction with our PNS membrane dynamics model. Specifically, from the original Zygote nerve atlas (Fig. 4a), we extracted paths of the peripheral nerve bundles as 3D curves with labels at their three types of phylogenic points; starting point, end-point, and any branching point as shown in Fig. 4. The starting point of the peripheral nerve fibers was the central nervous system (CNS), most frequently the spinal cord and the end-point was typically a distal extremity.

We define “nerve tracks” as individual nerve bundle segments between any of the phylogenic points identified in the previous step. The model contains approximately 1600 nerve tracks. The nerve tracks between the phylogenic points are represented in 3D by *centerline* curves that follow the center of the surface mesh of each nerve track. This was achieved by first applying a so-called *geodesic algorithm* that computes the shortest path along the edges of the surface mesh that connects the two phylogenic points. The resulting 3D curve runs along the surface of the mesh (i.e., on the surface of the “nerve tube”). In order to obtain the nerve centerline from this (i.e., the curve running along the center of the “nerve tube”), we first voxelized the entire nerve tree at 0.1 mm resolution and computed the distance measure of this voxelized nerve tree. The distance measure specifies, for each voxel, the distance to the nearest surface of the nerve tracks. Note that the distance measure is greatest for the center points of the nerve tube. The *centerline* curve was generated from these center points. Finally, we fit the resulting 3D curve with free-knots B-spline to avoid excessively high (unrealistic) curvatures of the nerve tracks. The final nerve atlas after this processing is shown in Fig. 4b, which has been color-coded by the number of branch points between the nerve track and the CNS.

### Nerve Membrane Model

**Figure 5:**
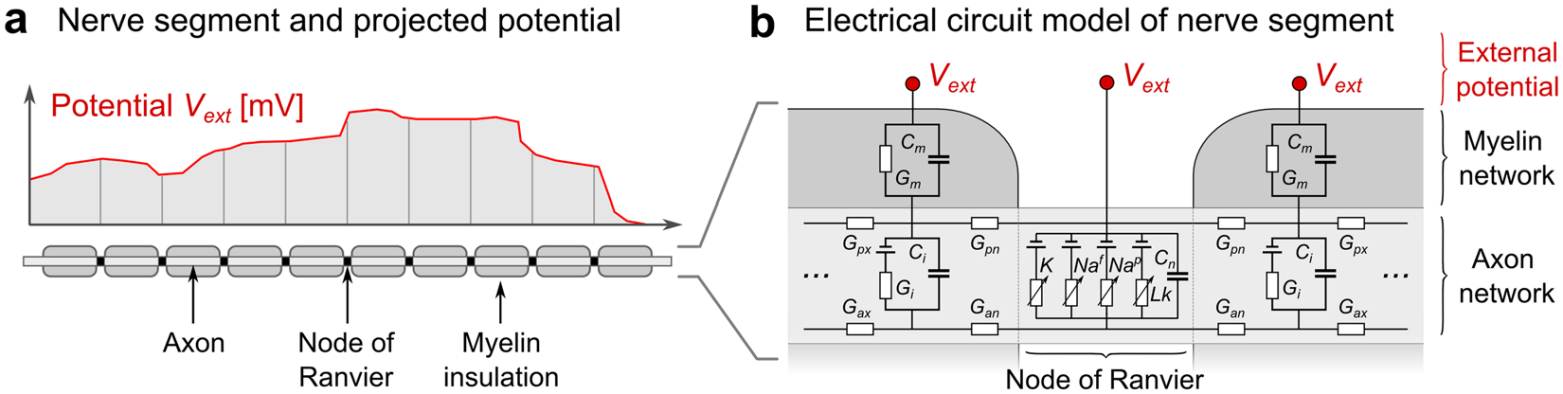
(**a)** Schematic of the discretization of the potential V_ext_ along the nerve segment with respect to the nodes of Ranvier. V_ext_ is determined from integrating the induced electric field calculated in Step 2. Node spacing is assigned based on estimated fiber diameter. (**b)** Electrical equivalent circuit for the MRG nerve model on each side of a node of Ranvier. Action potentials are created when the transmembrane potential change becomes greater than approximately 20 mV.

The electric fields calculated in the body model were projected onto the nerve fiber path and integrated to obtain potential differences along the fibers. These spatially varying potentials *V*_***ext***_ were modulated by the driving waveform of the coil and fed into the circuit model with myelin and node of Ranvier parameters. The McIntyre-Richardson-Grill model (MRG)^37, 38^ was used to simulate the response of the nerve to the voltage potentials induced along the fiber by the external fields. The MRG electrical nerve model is based on a double-cable representation of the myelinated axons (the inner cable models the axon, the outer cable models the myelin insulation sheath). Electrically speaking, these cables are connected in parallel, as shown in Fig. 5. The MRG model, unlike simpler models like the Hodgkin-Huxley Model^39, 40^ or the SENN model^41–43^, explicitly models action potential (AP) initiation. The effect of the local myelin insulation sheath on the excitability of the nerve is explicitly included, making this model more appropriate for simulation of PNS, since peripheral nerves are heavily myelinated. Since the myelin sheath insulates the axon, the nerve is most sensitive to the induced electric field at the locations of the nodes of Ranvier where the axon is in direct contact with the extra-cellular space. Each of the three nerve compartments (axon, myelin, and node of Ranvier) is modeled by RC-circuits with capacitance and resistance properties specific to each compartment (for the exact electrical parameters, see McIntyre *et al.* ^37^). In order to be able to simulate action potential initiation, the RC-circuit model of the nodes of Ranvier compartments includes non-linear terms, implemented as voltage-dependent resistances that model the sodium and potassium ion channels. This means that once the nerve fiber is strongly depolarized, the membrane becomes permeable for sodium and potassium ions (in the electric model this is represented as a small resistance), which leads to a large current flowing through the membrane and thus to an action potential. The different RC-circuits are connected via conductances that specify the transaxial and axial conductivity of the nerve. This allows modeling of the propagation of action potentials along the nerve fiber at realistic conduction velocities (approximately 25 m/s to more than 100 m/s, depending on the axon diameter).

The MRG electrical circuit is mathematically represented as a set of coupled differential equations. The input to the model is the external electric potential changes *V*_***ext***_ imposed by the external coil. In this work, we compute these potentials using electromagnetic simulation and by interpolation of the resulting electric fields along the nerve fiber paths. The output of the model is the resulting transmembrane potential change over time at each compartment of the nerve fiber model. These transmembrane potential changes are analyzed to decide whether or not an action potential has been initiated.

Each track of our nerve atlas was tagged with information about local nerve fiber diameters that we obtained from the literature^44^. These fiber diameters were used to feed the correct parameters into the MRG nerve membrane model. For example, in Table 1 of their paper, McIntyre *et al*.^37^ provide the model parameters of the MRG model which vary with axon diameter for 9 different diameters (ranging from 5.7 μm to 16.0 μm). This includes the thickness of the myelin insulation sheath that increases with fiber diameter. Stated conversely, the conductance of the myelin compartment decreases with fiber diameter. The spacing of the nodes of Ranvier also increases with the fiber diameter, from 0.5 mm for a 5.7 μm fiber to 1.5 mm for a 16.0 μm fiber. Together, the increase in both the myelin sheath thickness and node of Ranvier spacing with nerve diameter result in much higher excitability for large diameter fibers compared to smaller ones. Additionally McIntyre *et al*. provide fixed parameters (non-varying with diameter) in Table 2 of their paper^37^. To provide a full set of membrane model parameters for each nerve track in our nerve tree, we assigned nerve diameters based on the type of nerve fibers present in the nerve track. If any motor nerve fibers are present, we assumed large fiber diameters of 16 μm. For nerve tracks that only consist of sensory nerves (e.g., the digital nerve branches in the fingers, the sural nerve in the calf, or the posterior cutaneous nerve in the shoulder), we assigned smaller nerve fiber diameters (10 μm). Autonomous nerve bundles (e.g., those controlling the digestive or cardiovascular system) usually contain very small fibers (in the order of 2 μm diameter). Note that we are still refining the nerve fiber properties of our nerve atlas, as more detailed information becomes available (e.g., via studies of nerve conduction velocities that can be correlated with the fiber diameter).

### Generation of PNS Threshold Curves

**Figure 6:**
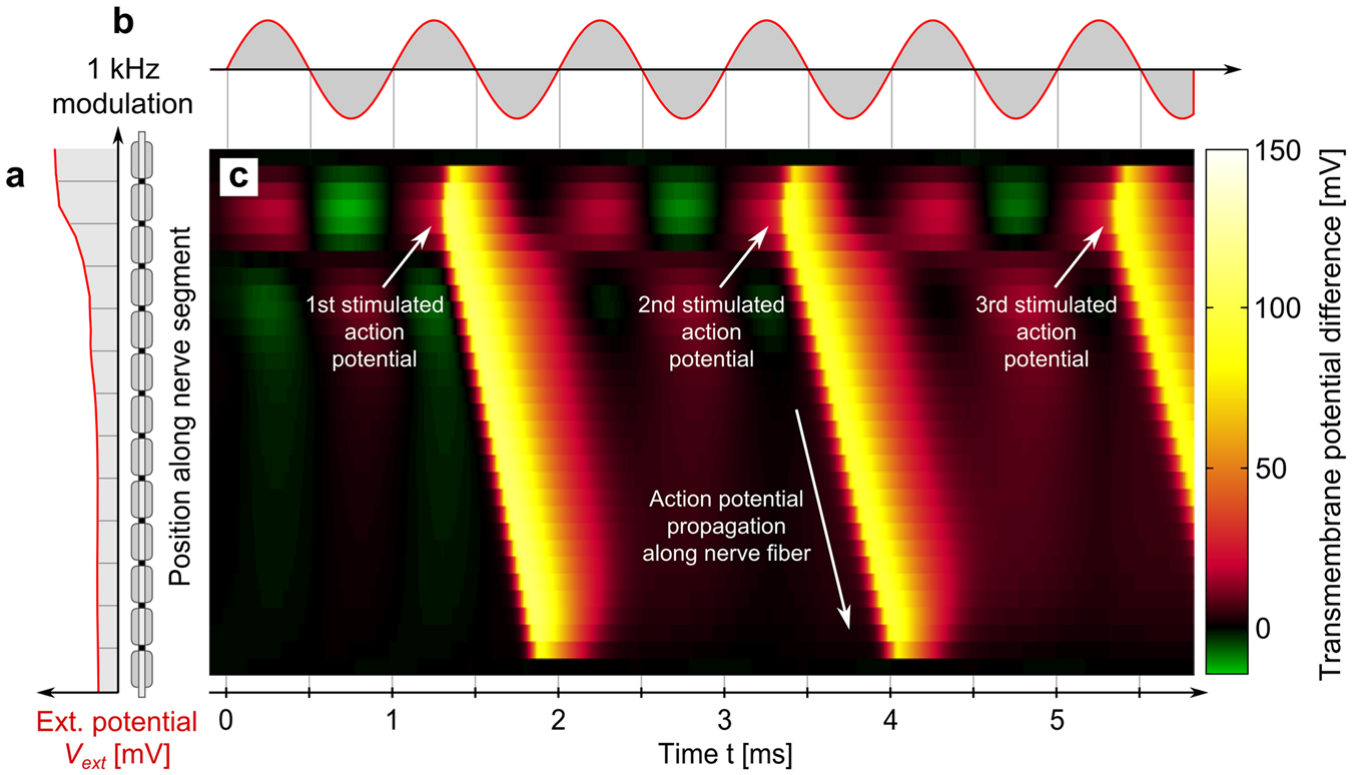
Exemplary evaluation of the nerve membrane model. (**a)** Potential created by coil along the nerve segment (V_ext_). (**b)** Time modulation of V_ext_ (1 kHz sinusoid at 125% of the PNS threshold). (**c)** The transmembrane potential as a function of space (position along the nerve, vertical axis) and time (horizontal axis) showing the creation of an action potential which then travels with a fixed velocity in space.

**Figure 7:**
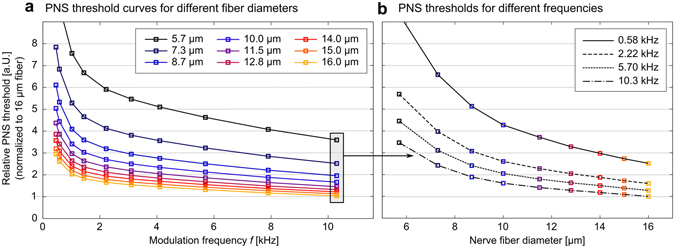
(**a)** PNS thresholds as a function of the frequency of the applied coil current (sinusoidal current) for different fiber diameters. The largest fibers show the lowest threshold (i.e., they are easiest to stimulate). (**b)** PNS thresholds as a function of fiber diameter, while keeping the frequency constant.

**Figure 8:**
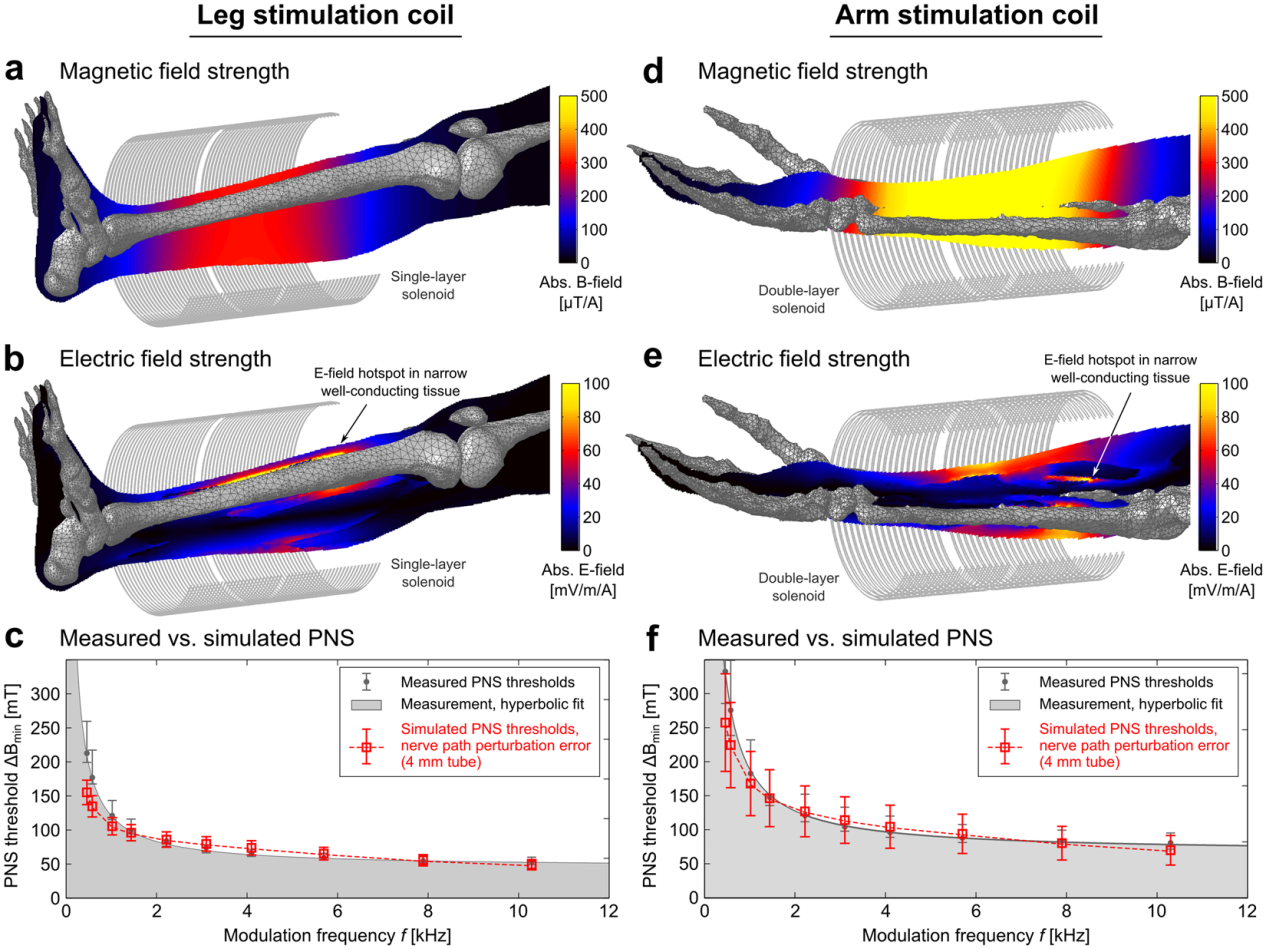
Calculated PNS thresholds as a function of coil current frequency for a leg coil and an arm coil compared to previously published experimentally measured thresholds^49^. In **c** and **d** we plot the measured and simulated PNS thresholds (amplitude of applied B-field which creates an action potential) as a function of coil current frequency. The error bars in the simulated PNS thresholds (red boxes) derive from the nerve path sensitivity analysis. Namely they represent the standard deviation of the thresholds over the 20 segments simulated within the 4 mm tube around the atlas nerve center.

The PNS threshold is defined as the smallest external field modulation strength that initiates an action potential. The PNS threshold depends on the frequency of the external electromagnetic excitation, the coil geometry, and the subject’s anatomy, which modulates both the strength of the electric field and its orientation with respect to the nerve fiber. To compute the PNS threshold curve, we modulated the spatial map of electric potentials (output of the electromagnetic simulation) interpolated along the nerve fiber by a sinusoidal waveform of different frequencies ramping the amplitude up for each frequency. We recorded the amplitude B_**min**_ that initiated an AP. This titration process is depicted in Fig. 6. After finding the threshold that initiates an AP for a given coil and body position, we plot the PNS threshold as a function of the excitation frequency. Calculated PNS threshold curves are shown in Figs. 7 and 8.

Processing the entire nerve tree at once is infeasible for a number of reasons. A single run of the MRG model requires solving a large set of coupled differential equations and is computationally expensive (simulation of the MRG model was performed within the NEURON environment^45^). The computational complexity increases with the total length of nerves in the model (i.e., with the number of nodes of Ranvier). For the leg coil, approximatively 8.6 meters of nerve fibers need to be analyzed (resulting in approximatively 5700 nodes of Ranvier and 63000 electrical compartments). For the arm simulation, there are 10.3 meters of nerve fibers resulting in 6900 nodes of Ranvier and 75900 electrical compartments. When solving the underlying differential equation, we used a time step of 0.5 μs for a 1 kHz coil current waveform and decreased this value linearly for higher frequencies (i.e., independently from the frequency every bipolar pulse is resolved by 2000 time points). Thus, a smaller time step was used compared to other nerve model simulations. For example McIntyre *et al*. suggested that (using a block pulse to stimulate the model) a time step of 1 to 5 μs would accurately solve the differential equation of the MRG neurodynamic model. Our analysis of the convergence of the solution to the differential equations underlying the MRG neurodynamic model suggested a smaller time-step was needed. We do not know if this difference was derived from a more conservative convergence criterion or reflects a more complicated temporal dynamics within the nerve model for our situation. The stimulus consisted of 15 sinusoidal periods, resulting in durations of 32.6 ms to 1.5 ms for coil current frequencies of 0.46 kHz to 10.3 kHz. Note that based on these parameters, calculation of PNS threshold curves using the entire nerve tree would require at least a week of computation time (assuming that no parallelization is used). Processing the entire nerve tree also results in a high computational overhead. For example, if a nerve track branches into a major track and a minor track, the part of the nerve prior to the branching point will be simulated twice: once as being part of the major track and once as being part of the minor track (unless the nerve track is divided appropriately).

To increase computational efficiency, we divided the nerve tree into smaller sub-segments without any branches, where each sub-segment has a reasonable length (ideally less than 100 nodes of Ranvier). We refer in this work to these sub-segments of the nerve tree as *nerve segments*. Dividing the nerve tree into smaller nerve segments also means dividing the underlying differential equation, which may cause numerical instabilities and artefacts at the boundaries of the nerve segments. Therefore, we implemented methods to identify the locations of the nerve tree, where dividing the nerve fiber into multiple nerve segments only has a mild effect on the model accuracy and, therefore, on the resulting PNS thresholds. Rattay and others^46, 47^ showed that the second spatial derivative of the extracellular potential along the fiber can be considered the main determinant (also referred to as “activation function”) of nerve stimulation. This metric is often used to guide the development of electromagnetic coils for neuro-stimulation or neuro-modulation devices^48^. As a consequence, an electric potential pattern that varies only linear along the fiber does not generate an activation function (no stimulation of the nerve). Instead the electric potential leads to a constant current flow along the fiber without any current flowing through the membrane. This means that the external electric field is not effective at depolarizing the membrane and the nerve segment will not initiate an action potential in this region. Thus these regions can be safely ignored when searching for the PNS threshold. We found that dividing the nerve tree into nerve segments at these locations only has negligible effects on the excitability of the nerves and, therefore, on the AP thresholds. After defining the nerve segments (we simplified the nerve model to 304 and 359 nerve segments for the leg and arm stimulations, respectively) we further reduced computation time by solving the MRG model only for those segments where the electric potential leads to a substantial flow of current through the membrane (possibly leading to an AP). Note that the definition of individual nerve segments also allows for a heavy computational parallelization since every nerve segment can be excited and simulated independently.

### Preliminary Sensitivity Analysis

In order to estimate the sensitivity of the PNS threshold curves to the nerve atlas (i.e., to the path of the nerve segments relative to other tissue structures), we have conducted a preliminary sensitivity analysis. The nerve segments are particularly sensitive to spatial deformations that alter the curvature of the segments (e.g., only applying shifts to a nerve segment within a linearly varying electric potential does not influence the stimulation threshold). In order to implement a spatial uncertainty of the nerve segments path, we defined a tube of a certain radius (2mm and 4mm) following the nerve segment and allowed the nerve to traverse the tube along a flexible path. This is achieved by using a 3D Gaussian deformation field: for each spatial dimension we randomly distributed 10 three-dimensional Gaussians within the bounding box of the nerve segments (overall 30 Gaussians). The standard deviation of the Gaussians (2 cm in this example) determines the smoothness of the deformation; the function value of the sum of Gaussians determines the local deformation strength along the different axes. The weighting of the 30 Gaussians was chosen randomly such that 1) the deformed nerve segment remains within the tube (this limits the maximum local deformation), and 2) the segment does not intersect the bone tissue or outer hull of the body (for example for superficial nerve segments). We generated 20 deformed nerve segments for tube radii of 2 mm and 4 mm and computed PNS threshold curves for each segment. We then use the standard deviation of the PNS thresholds over the 20 segments as means of the sensitivity of the predicted threshold curves to the path of the nerve segment.

### Validation against Experimentally Measured Thresholds

We compared our simulated PNS threshold curves with those measured experimentally using leg and arm solenoid coils applied to healthy volunteers^49^. The two coils were simulated based on their described dimensions and geometry using CST’s frequency domain solver which uses a low-frequency approximation of Maxwell’s equations. The coils were modeled using infinitely thin, perfect electrical conductor current paths. Boundaries were modeled as a box of perfect electrical conductors (PEC) enclosing the entire simulation domain 50 cm away from the tissue model or the coil (low-frequency FEM solvers usually require PEC boundary constraints). We found that this PEC boundary spacing is reasonable for these two simulation setups and that increasing the boundary spacing to 500 cm altered the tissue electric field maps by only about 1–2%. The experimental comparison data was taken in an open setting (approximately “free space”). But we note that MRI gradients are used within the more complex conducting environment of the magnet and its cryostat, which might need to be taken into account for MRI gradient studies. The simulated leg coil consisted of 54 turns in a single layer (length of 24 cm, diameter of 19 cm); the arm coil consisted of 72 turns in two layers (length of 17 cm, diameter of 11 cm). The body model was placed inside the coils to mimic the experimental setup as closely as possible, although only the photo in the published paper provided guiding information. In the simulations, only the leg (until 30 cm above the hip joint) and the arm (including the shoulder joint) were simulated as the effects of the coils are limited to these regions.

### Sinusoidal vs. Trapzoidal Waveforms

MRI gradient encoding most often uses trapezoidal waveforms where electric field generation is confined to well-defined pulses during the ramps of the trapezoid. It is well documented that for a given peak B-field strength, the trapezoidal waveform has increased PNS thresholds compared to a sinusoidal waveform^50, 51^. Furthermore, it has been observed that the PNS thresholds increase linearly with the pulse duration (ramp rise-time)^3^. In order to determine if our model captures these two observations, we applied both sinusoidal and trapezoidal waveforms using ramp rise times relevant to MRI (from 0.1 to 1.0 ms). The PNS thresholds (applied solenoid B-field amplitude) as a function of pulse duration were assessed in terms of linearity with a linear fit and in terms of the chronaxie times (i.e., the slope of the linear fit).

## Results

Figure 6 shows the time evolution of the membrane potential for a 16 μm fiber diameter in response to an applied external waveform. Figure 6a shows the external electric potential imposed by the coil at the different nodes of Ranvier. This spatially varying electric potential pattern is modulated in time by the 1 kHz sinusoidal waveform shown in Fig. 6b. The resulting membrane dynamics are shown in Fig. 6c: in this 2D plot, the vertical axis specifies the spatial location along the nerve segment and the horizontal axis specifies time. During the first two sinusoidal half-lobes, the nerve segment is alternatively hyperpolarized (green) and depolarized (red) but only the third half-lobe initiates an action potential (yellow) in the upper part of the segment. This action potential then propagates along the nerve segment without significant further perturbation by the external electric potential’s waveform. After this first action potential the nerve membrane recovers (this is the refractory period during which the ion-channel pumps are active and no action potential can be evoked) and a second and third action potential are initiated by the 7th and 11th sinusoidal half-lobe, respectively.

Figure 7a shows PNS threshold curves for a nerve segment in the leg for varying values of the fiber diameter (5.7 μm to 16.0 μm). The field modulation strength (vertical-axis) is normalized to the PNS threshold for the largest fiber (16.0 μm diameter) at the 10.4 kHz modulation frequency. These PNS threshold curves coincide with experimental observations^52, 53^ and theoretical predictions^24, 43, 54^ that nerve fibers with a large diameter are more easily excited than smaller fibers. The threshold for the smallest diameter fiber is about 3.5 fold that of the largest fiber simulated (16.0 μm). In Fig. 7b, we show PNS thresholds as a function of fiber diameter, for given constant frequencies of the applied fields. Note that the PNS thresholds change very quickly for small fiber diameters and rather slowly for large fibers.

Figure 8 shows the PNS simulation thresholds as a function of applied field frequency for the leg and arm coils. The electric field magnitude is shown overlaid on the anatomical model. Figure 8 also shows the resulting simulated PNS threshold curves as well as the measured thresholds from Saritas *et al*.^49^. The measured values represent the median over 26 subjects (error bars indicate 25th and 75th percentile). The B-field generated by the modeled coil agrees well (within 1%) with the experimental value (measured values: 214 μT/A for the leg coil, 410 μT/A for the arm coil, simulated values: 211.86 μT/A for the leg coil, 407.1 μT/A for the arm coil). The electric field maps show that the electric field patterns are significantly modulated by the anatomical details of the model. Electric field hotspots occurred in tissues with low conductance, such as layers of fatty tissues separating different muscle fiber bundles and in regions where the conductivity is significant but the geometry narrows (e.g., the tibialis anterior muscle whose width becomes very small close to the shin bone region, arrow in Fig. 8b). Although the average measured PNS thresholds (gray region) from the 26 subjects and the simulated PNS thresholds (red curve) are in good overall agreement, there is some deviation in the low-frequency region of the PNS curves for both the leg and arm stimulations. However, the experimental values also exhibit increased variance in this frequency range. We repeated the PNS simulations using a range of spatial deformations applied to the most sensitive nerves, allowing the nerve segments to vary freely within a tube surrounding the unaltered nerve segment. For a tube radius of 2 mm (4 mm) the PNS thresholds computed for 20 deformed nerve segments in the arm showed a standard deviation of 15% (30%). The leg example showed an uncertainty of 6% and 14% for the 2 mm and 4 mm tube, respectively.

**Figure 9:**
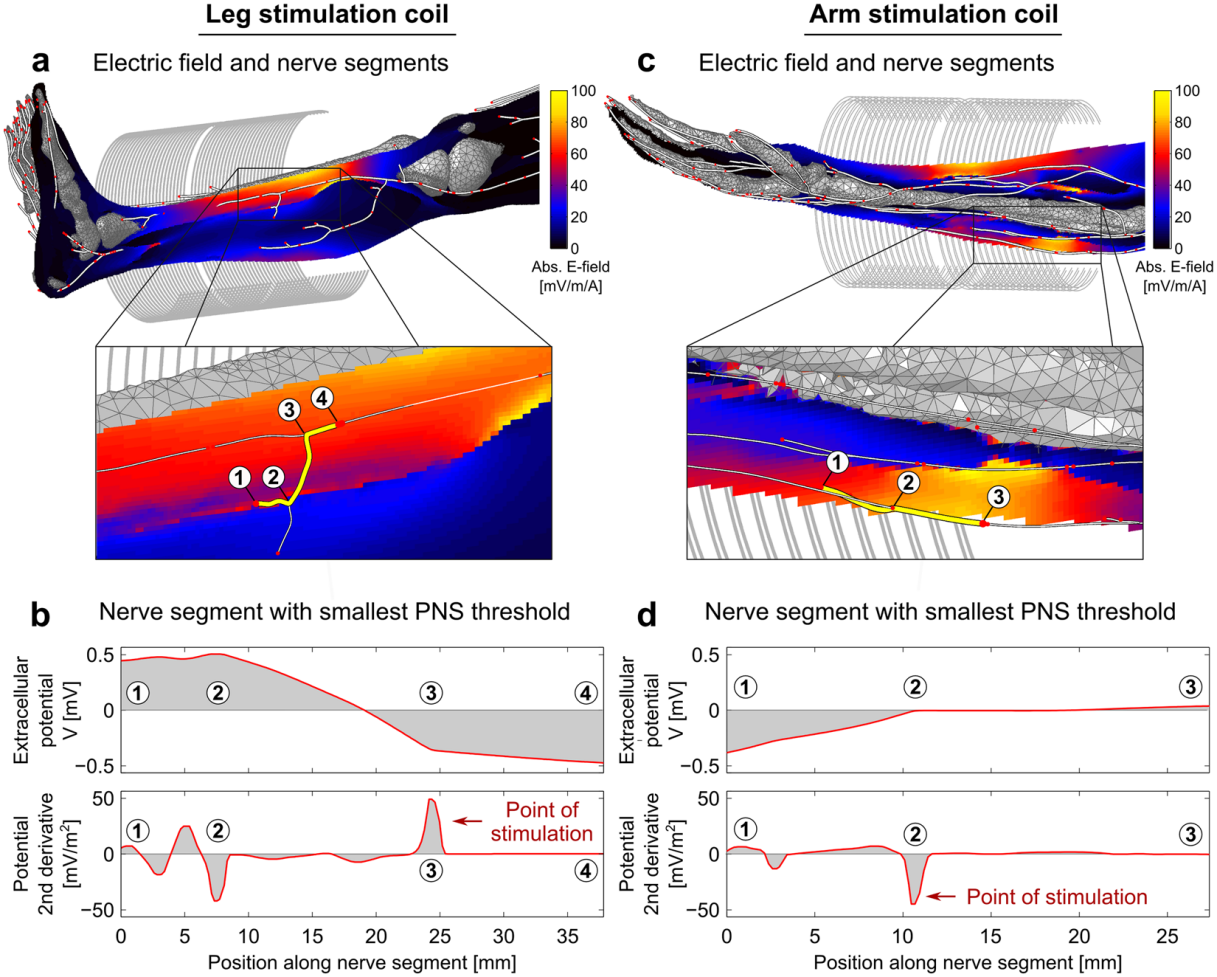
Simulated nerve segments (red dots indicate end points of each nerve segment), superimposed onto the coil configurations and E-field maps (**a** and **c**). In the zoomed images, the nerve segments with smallest stimulation thresholds are depicted. In **b** and **d**, we show, for these nerve segments, the external electrical potential and its second derivative (which is proportional to the current flow through the membrane).

In Fig. 9 we illustrate the coil configurations and E-field maps, overlaid by the simulated nerve segments (red dots indicate boundaries of the individual nerve segments). In the zoomed images, the nerve segments with lowest stimulation thresholds are shown (these segments determine the PNS threshold curves shown in Fig. 8). The bottom row of Fig. 9 shows the electric potential and its second derivative of these two nerve segments (this is proportional to the amount of current entering of leaving the membrane^46, 47^). The kinks of these nerve segments in high E-field regions (positions 2 and 3 at the left, position 2 at the right) cause a sudden change in slope of the electric potential along the nerve segment, leading to a large inflow or outflow of current (peaks in the second derivative of the electrical potential). In both the leg and arm situation, the stimulated nerve segments pass through a rather high E-field region (maximum E-field strengths along the excited segments were 66.6 mV/m/A in the leg and 47.7 mV/m/A in the arm). However, it is the high curvature of these nerve segments that plays a major factor in making these segments particularly sensitive to the applied E-field.

**Figure 10:**
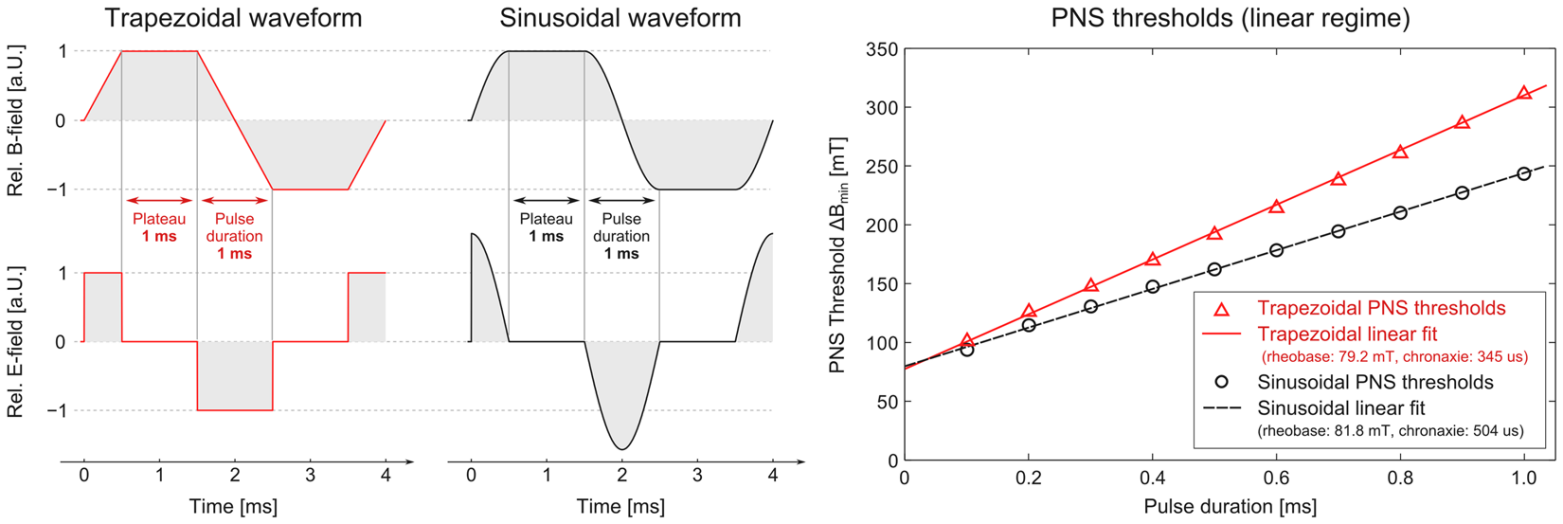
Trapezoidal and sinusoidal B-field waveforms and the resulting E-field pulses (plateau duration 1.0 ms, pulse durations between 0.1 and 1.0 ms, overall 10 bipolar pulses) and the resulting PNS thresholds as a function of pulse duration (i.e., in the linear regime).

Figure 10 shows the trapezoidal and sinusoidal waveforms studied and the pulse durations during which dB/dt (and thus an electric field) is present. Figure 10 also shows the PNS thresholds for the arm coil as a function of pulse duration as well as the linear fit. The MRG neurodynamic model used in this work reproduces the increase of the PNS thresholds for trapezoidal pulses. The linear fit of the sinusoidal (R² = 0.9913) and trapezoidal thresholds (R² = 0.9947) reveals chronaxie times of 504 μs and 345 μs, respectively, which is in very good agreement with experimental data^18^.

## Discussion

In this work, we presented a simulation framework for estimating the PNS thresholds for electromagnetic coils such as an MRI gradient coil or MPI drive coil operating in the kHz-range. In the first step, we calculate the electromagnetic fields created by the coil in a realistic body model, as has been reported in numerous previous studies. We then model the effect of these fields on realistic nerve models, including the relative geometry between field and nerve. The full body peripheral nerve model incorporates the properties of the myelinated fibers allowing the non-linear effect of the fields on membrane potentials to be calculated. The modeled time-dependent waveform is increased in steps to identify the lowest applied field (and thus current amplitude) which initiates an action potential. The location of the stimulation can also be recorded. We validated this process by comparing to the published experimental PNS threshold curves for two solenoid coil geometries.

The PNS simulation method achieved a reasonable agreement with the experimental PNS threshold curves, although the lower frequencies studied (0.5 kHz to 1 kHz) had some deviation from the experiments. In this frequency range the threshold is changing very rapidly with frequency and the experimental data has a relatively high inter-individual variance, suggesting increased variance with patient anatomy. For the MPI applications, the relevant frequency range will likely be above this range (e.g. above 10 kHz), suggesting a good possibility for accurately predicting thresholds. For MRI, the 0.5 kHz to 2 kHz is a relevant range for stimulation by the readout gradient. For example, EPI gradient readouts are generally in the 0.75 kHz to 1.5 kHz range. Nonetheless, even a relative prediction between two coil design models can inform the gradient coil optimization process. Furthermore, we believe that the accuracy of the predicted PNS thresholds in the low kHz-range can be improved by using multiple body models (with matching nerve atlases) of different ages, body-mass indices, and genders. At this stage, our calculation of the PNS threshold was performed for a single female body model and does not yet include estimation of the uncertainty due to the normal biological and anatomical variability of patients undergoing MRI and MPI scans. Experimental results show that the standard deviation across individuals for PNS can be on the order of 40%^6, 49^, which requires the generation of additional body models, ideally capturing the anatomical variability of the subject population as well as slight differences in placement of the subject within the coil. Current use of experimentally determined thresholds is limited to population average thresholds for a given MRI gradient coil design in setting regulatory limits^55^. We envision our simulation determined thresholds to initially be used in a similar way; to mimic population averages with a small number of body models to allow comparison between MRI gradient designs during an iterative optimization. Full characterization of the population variance will require extensive work and patient-specific modeling of PNS is likely even further off in the future.

The experimental data obtained by Saritas *et al*.^49^ to which we compare our simulated thresholds, possessed a threshold variation over the studied individuals (25th to 75th percentile) of about 30% to 40% for the arm stimulation case and 28% to 20% for the leg stimulation (at 460 Hz and 10.6 kHz, respectively). The results of our preliminary sensitivity analysis to deformations of the nerves relative to other tissues in the model indicate that the model is sensitive to the nerve shape and location relative to other tissues to a level similar to the variability of the experimental data. Note that there are other sources of variability expected for the estimation of PNS thresholds such as variations in the placement of patients in the coil (non-biological), as well as electromagnetic tissue properties *in vivo* (biological). Another deviation between the simulated and experimental PNS threshold curves are their slightly different shapes (when plotted as a function of frequency). This is likely due to the fact that there are aspects of action potential initiation and propagation that are not yet fully captured by the MRG membrane model. The MRG model is, however, currently one of the most sophisticated nerve models available. The procedure can be updated when more detailed membrane models become available, and might also prove a useful tool for refining these models.

Recordings made by Saritas *et al*. during the stimulation experiments indicate that the most prominent sites were the proximal forearm and the proximal tibia region, i.e., roughly at the proximal ends of the two stimulation coils (personal communication with Emine Saritas, March 12, 2017). Both motor and sensory sensations occurred in the experiments. In our arm simulations, stimulations originated in a branch of the radial nerve that (among others) innervates several muscles at the proximal forearm close to the elbow joint (including extensor carpi radialis longus muscle, extensor carpi radialis brevis muscle, supinator muscle and others). For the leg simulation, the AP first occurred in a sensory nerve: a branch of the saphenous nerve in the proximal tibia region. Although there is reasonable agreement between the site of stimulation reported in the experimental study and simulation results, it is difficult to be sure that the model identified the same nerve segment experience by the human subjects.

Only modest experimental data is available on the quantitative relationship between the excitability of a nerve fiber and its axon diameter, making it very hard to verify whether the used MRG neurodynamic model correctly describes this relationship. However, from the neurodynamic modelling perspective it is well established that for very small fiber diameters (in the order of 1 μm) the stimulation threshold is proportional to the “inverse square” fiber diameter. When increasing the fiber diameter up to 20 μm this relationship progressively approaches an “inverse square root” relationship^43^. This behavior can be verified by a logarithmic plot of the threshold curves shown in Fig. 7b and evaluating the slope of these curves at different fiber diameter points (for example, in the logarithmic plot a slope of −2.0 indicates an “inverse square” relationship, a slope of −0.5 an “inverse square root” relationship). In our simulations, we found a slope of −1.4 at 5 μm and −1.1 for 16 μm which is consistent with previous observations (a slope of −2.0 at 2 μm and a slope of −0.5 at 20 μm, with a continuous progression for intermediate diameters). However, some deviation is expected due to the effect of the myelin insulation which is not explicitly modelled in the other neurodynamic models. We rely on these previously published results to insure that the MRG neurodynamic model satisfactorily describes the dependence of PNS threshold on fiber diameter.

In this work we define the PNS threshold as the field modulation strength where the first AP is initiated in any of the nerve segments in the simulated part of the body model. In fact for very sensitive areas of the human body (skin, lips) it is well known that even a single AP in a single sensory nerve fiber already produces a sensible perception^56^. Although for motor nerves (which are the nerves that are first stimulated in the arm solenoid), a single AP in a single nerve fiber may not produce a sensible muscle contraction. Nonetheless we argue that the PNS threshold for single AP generation is very close to the threshold for a perceivable sensation for two reasons. Firstly, motor nerves possess a rather short refractory period in the order of 2 ms, allowing for a repetition frequency of about 500 Hz (the refractory period decreases when increasing the fiber diameter, meaning that motor nerves achieve a higher AP repetition frequency than sensory nerves^57^). Since our stimulation pulses usually have the same or higher frequencies (460 Hz to 10.6 kHz), the stimulation pulse at the PNS thresholds will generate multiple APs, one after each recovery cycle (roughly every 2 to 3 ms^58^, cf. Figure 6). Secondly, for healthy subjects the fiber diameter distribution of motor nerves in a single nerve fiber is rather sharp meaning that the different nerve fibers possess very similar stimulation thresholds^44^ (as opposed, e.g., to patients suffering from Multiple Sclerosis, where demyelination of the nerve fibers causes of broader distribution of the stimulation thresholds). Along with the fact that the entire nerve fiber bundle can be considered to experience the same local electric field, this means that the threshold for the very first AP in a single fiber (which may not be perceivable, especially for motor nerves) and the threshold for initiation of a larger number of APs in various fibers of the same nerve bundle (causing a perceivable sensation) should only differ mildly.

We found that accurate modeling of the PNS threshold curves required very careful definition of the “nerve segments”, which we use in this work to split the whole-body nerve tree into computationally manageable sections. For example if an external electric field stimulates the nerve segment at the periphery of the nerve segment (as opposed to the central nodes of Ranvier of the segment), this often leads to an underestimation of PNS thresholds. On the other hand, if nerve segments are defined with knowledge of the electric field in order to guarantee that the strongest electric potential change always occurs at the center of a segment, accurate prediction of AP initiation is possible. We validated this approach by comparison of PNS stimulation of very long nerve segments.

We have found that the nerve membrane dynamics are rather sensitive to even small anatomical details of the body model. In fact, small structures, especially bottle-necking structures, may have a more prominent impact on the electric field hotspots than large, uniform structures. In our simulations the largest E-field hotspots often occurred in regions where a well-conducting tissue geometry narrows (as highlighted in Fig. 8b and e). Additionally we found that thin layers of low conductivity tissues (like fatty tissue) enclosed by high conductivity tissues (like muscles) may act as a capacitance creating high E-fields in the insulating layer. If a nerve fiber passes through these regions, this might facilitate stimulation. However, we have not yet quantified the effect of the level of detail of the body model on the accuracy of the PNS threshold curves. Ideally we would like to simplify the body model as much as possible (to decrease computation time of the EM field simulations), while ensuring accurate PNS threshold curves. Additional investigations are needed to determine the most important properties of the body model such as the required number of tissue classes, distribution of fatty tissue, or spatial properties of the tissue objects (we note that for the skin in our model we used a rather large thickness of 3mm, which significantly simplified meshing).

The detailed nerve atlas was, of course, central to our simulation. PNS is modulated by the relative angle of the imposed electric field to the nerve fiber. Thus the PNS effect is different from scalar parameters such as Specific Absorption Rate (SAR) which quantifies energy deposition from the much faster oscillations of the applied radio frequency (RF) field which are in the MHz range. The electric fields responsible for SAR are too fast for the nerve membranes to respond to, and simply cause heating of the electrically lossy tissue. Thus SAR is determined by the strength and duration of the applied radiofrequency fields, the magnitude of the induced E-fields and the conductivity of the tissue. SAR is relevant at considerably higher frequencies than considered in our simulation. Roughly speaking, SAR becomes the dominant concern for applied fields above 5 MHz. In contrast, PNS is the dominant concern for the oscillation frequencies of approx. 500 Hz to 10 kHz studied here. Unlike SAR where only the magnitude of the E-field matters both the strength and direction relative to the nerve segment determine the local PNS threshold. This, together with the observed electric field hot-spots, means that it is critical to know the exact locations and orientations of the nerves with respect to the rest of the body model. The information about location and orientation of the major nerve fibers is encoded by our nerve atlas and no manual user input of the nerve fiber properties or location/orientation is required beyond classifying the segment as motor, sensory or autonomous for which a diameter assignment of 16 μm, 10 μm or 2 μm was made (another source of fiber diameter information is measurement of the nerve conduction velocity that depends linearly on the fiber diameter, however, these studies are limited to a few major nerve tracks like radial, sural, tibia nerves^12^). The detailed nerve properties for each of these diameters are then dictated by McIntyre *et al*.^37^. Nonetheless, experimental threshold measurements show variance across individuals suggesting that the use of multiple body models may be useful to get a more complete simulation of the range of responses. The experimental variance might also result from variations in body position within the coil. The relative importance of these two sources of variance could be assessed from studies repositioning an individual subject and noting the variance of the thresholds. Finally, while the magnetic field produced by the modeled solenoid coils is uniform, the E-field pattern is far from uniform. This underscores the importance of the geometry of the conductive tissues in shaping the current flow, thus the electric field is in contrast to the near transparency of the body to the magnetic field at these frequencies. It also suggests the importance of the nerve model accurately reflecting nerve locations within the tissue.

The simulations carried out in this work (Fig. 7) are consistent with previous physiological experiments^52, 53^ and theoretical investigations^24, 43, 54^ showing that nerves with larger fiber diameters are excited more easily than smaller ones and, therefore, tend to determine the PNS threshold. This is fortunate for our efforts since it means we do not need to model the smallest fibers. In the human body, the largest fibers are those that innervate the neuromuscular spindles, so-called A-α fibers, with fiber diameters of about 10–20 μm and conduction velocities of up to 120 m/s^59^. Muscular spindles are motor receptors that detect information about length changes of a muscle. The associated nerves transmit this information to the CNS that initiates a reflex in terms of a muscle contraction (such as the patellar reflex or “knee reflex”). This phenomenon is often observed in MRI experiments where PNS sensations in the extremities are experienced as reflex-like muscle contractions. It seems to be a reasonable assumption, that any nerve bundle that contains motor nerves also contains at least a small fraction of these large nerve fibers. The AP generation observed at the PNS threshold for the arm case (Fig. 8f) resulted from stimulation of this kind of motor nerve fibers with 16 μm diameter. For nerve bundles that solely contain sensory nerves (such as in the fingers that do not contain muscle tissue), the largest nerve fibers are so-called A-β fibers whose diameter is in the range of 7–15 μm with conduction velocities of up to 70 m/s (in the leg case shown in Fig. 8c, a sensory nerve fiber was stimulated first). Unmyelinated nerve fibers such as the ones controlling the digestive system (so-called C-fibers, in the order of 1 μm diameter, up to 2 m/s conduction velocity) are hard to stimulate by external E-fields and, therefore, may be ignored in this context. The same holds for cardiac nerves, that possess an up to 200-fold higher stimulation threshold compared to motor fibers^60^. However, there is evidence^61^ that the thresholds for PNS and cardiac stimulation converge for long pulse durations. It would be interesting to see if an extension of our simulation framework could validate this effect, however, several changes to our model would be needed. The MRG neurodynamic model used in this work was designed for fiber diameters ranging from 5 to 16 μm and may not be suitable for simulation of cardiac nerves (having fiber diameters of approx. 1 μm). Thus other neurodynamic model approaches that are more appropriate to autonomous nerve fibers are needed^62, 63^. This, together with a more complete depiction of cardiac nerves than found in the Zygote model may allow the expansion of the simulation approach beyond motor and sensory nerves, and shed light on the relationship between cardiac and peripheral nerve thresholds.

A limitation of this study is the relatively simple coil geometries studied (compared to a full MRI gradient coil). The MPI solenoid coil produces a rather uniform magnetic field pattern whereas the gradient coil produces a first-order spatially varying magnetic field. Thus, the magnetic field of the gradient coil is more spatially complex than that of the MPI solenoid coils analyzed in this work. Nonetheless, the electric field in both cases exhibits a complex spatial pattern. We are currently working with an MRI manufacturer to provide access to both the detailed winding patterns and human subject threshold experimental results to allow us to validate our approach for these cases. Another direction for evaluation of our PNS simulation framework is to replicate and perhaps shed light on the experimental work carried out by Recoskie *et al*.^18, 64^ about differences in chronaxie times obtained by electro and magnetostimulation experiments.

In this work, we concentrate on introducing and validating our modeling framework. The degree to which this framework can improve MRI gradient or MPI coil design is an important open question. The introduced methods are, at best, necessary, but not sufficient for improving design and may not be better than existing approaches which note that PNS correlates well with volume of the linear region of the gradient for conventional designs^6^. It is therefore likely that simply modulating existing designs, such as scaling or mildly altering wire density will result in PNS threshold changes that are already described by the current PNS scaling arguments. To go beyond this, unconventional design approaches will likely be needed, especially those that introduce degrees of freedom which can be exploited in an optimization framework. To this end, we note that new approaches are starting to emerge, such as the recent PatLoc coil comprising 84 independently drivable loops^65^, that perhaps offer both the degrees of freedom and necessary departure from standard design, but currently this observation is unproven.

Despite the role PNS plays in limiting the application of kHz-range magnetic fields in MRI and MPI methods, relatively few tools exist to predict PNS thresholds based on a specific coil wire pattern. Instead proxies for direct prediction, such as the volume or length^6, 66, 67^ of the linear gradient region is used to inform design. In this work, we build on previous work simulating the relevant E-fields in the human body to attempt to predict stimulation thresholds and locations based on nerve and tissue geometry rather than coil metrics such as length or volume of the linear gradient region. We also build on previous work assessing how E-fields induced in the human body interact with a nerve segment, informed from dynamic nerve models. We add a general nerve atlas, labeled with the relevant nerve model parameters and in registration with the predicted local E-fields to be able to accurately predict PNS thresholds for experimental coil configurations.
